# Sporogenesis in *Physcomitrium patens*: Intergenerational collaboration and the development of the spore wall and aperture

**DOI:** 10.3389/fcell.2023.1165293

**Published:** 2023-04-13

**Authors:** Karen S. Renzaglia, Neil W. Ashton, Dae-Yeon Suh

**Affiliations:** ^1^ Department of Plant Biology, Southern Illinois University, Carbondale, IL, United States; ^2^ Department of Chemistry and Biochemistry, University of Regina, Regina, SK, Canada

**Keywords:** aperture, exine, intine, perine, *Physcomitrium patens*, sporogenesis, spore wall, tapetum

## Abstract

Although the evolution of spores was critical to the diversification of plants on land, sporogenesis is incompletely characterized for model plants such as *Physcomitrium patens*. In this study, the complete process of *P. patens* sporogenesis is detailed from capsule expansion to mature spore formation, with emphasis on the construction of the complex spore wall and proximal aperture. Both diploid (sporophytic) and haploid (spores) cells contribute to the development and maturation of spores. During capsule expansion, the diploid cells of the capsule, including spore mother cells (SMCs), inner capsule wall layer (spore sac), and columella, contribute a locular fibrillar matrix that contains the machinery and nutrients for spore ontogeny. Nascent spores are enclosed in a second matrix that is surrounded by a thin SMC wall and suspended in the locular material. As they expand and separate, a band of exine is produced external to a thin foundation layer of tripartite lamellae. Dense globules assemble evenly throughout the locule, and these are incorporated progressively onto the spore surface to form the perine external to the exine. On the distal spore surface, the intine forms internally, while the spiny perine ornamentation is assembled. The exine is at least partially extrasporal in origin, while the perine is derived exclusively from outside the spore. Across the proximal surface of the polar spores, an aperture begins formation at the onset of spore development and consists of an expanded intine, an annulus, and a central pad with radiating fibers. This complex aperture is elastic and enables the proximal spore surface to cycle between being compressed (concave) and expanded (rounded). In addition to providing a site for water intake and germination, the elastic aperture is likely involved in desiccation tolerance. Based on the current phylogenies, the ancestral plant spore contained an aperture, exine, intine, and perine. The reductive evolution of liverwort and hornwort spores entailed the loss of perine in both groups and the aperture in liverworts. This research serves as the foundation for comparisons with other plant groups and for future studies of the developmental genetics and evolution of spores across plants.

## Introduction

The evolution and elaboration of a novel diploid sporophyte generation was foundational to the terrestrialization of plants (Ligrone, et al., 2012). With the interpolation of this generation into a haplontic life cycle came the evolution of key signature features of land plants that include the embryo, placenta, and spores. Although the complexity of the sporophyte generation in the earliest land plants remains controversial, fossil evidence for land plant spores is not disputed and predates the appearance of plant megafossils by over 40 million years (Strother and Foster, 2021). In the earliest land plants, highly resistant single-celled spores served as the primary means for perennation and dispersal as they do today in seed-free bryophytes and pteridophytes.

The resilience of spores and their ability to persist through hundreds of millions of years of evolutional history are attributed to the existence of a complex spore wall that is impregnated with sporopollenin, a recalcitrant and enigmatic heteropolymer (Grienenberger and Quilichini, 2021). Despite the significance of spores in plant evolution and diversification, the origin and development of the spore wall is incompletely characterized for many model plants, including the moss *Physcomitrium patens*.

Aspects of spore development in mosses have been reported in diverse taxa, including *Sphagnum* (Brown et al., 1982), *Andreaea* (Brown and Lemmon, 1984), *Andreaeobryum* (Polevova et al., 2022), and *Takakia* (Renzaglia et al., 1997), the sister taxa to peristomate mosses. Among bryopsid mosses, sporogenesis has been examined in scattered taxa, including *Trematodon* (Brown and Lemmon, 1981), *Ditrichum* (Brown and Lemmon, 1980), *Timmiella* (Gambardella et al., 1993; Gambardella et al., 1994), *Archidium* (Brown and Lemmon, 1985), and *Amblystegium* (Brown and Lemmon, 1982). As intimated by Wallace et al. (2011), several critical questions regarding spore wall development in *P. patens* remain unanswered, including the derivation of sporopollenin-containing layers and aperture development.

The present study was designed to fill the gaps in the published work on spore ontogeny in *P. patens*, an important plant model system since the publication of its genome sequence (Rensing et al., 2008). We present detailed ultrastructural evidence that systematically identifies the process of spore differentiation and the iterative construction of spore wall layers following meiosis. Spore maturation is assessed in coordination with changes in the milieu in which spores develop, including the spore mother cell (SMC) wall and matrices within the capsule locule. We conclude that spore development in this moss involves an integrated coordination between the diploid sporophyte and haploid spore that systematically produces and lays down the characteristic spore wall, while supporting the expansion and maturation of the spore proper. This work provides a benchmark for the interpretation of data from research designed to elucidate the genetic and biochemical basis of sporopollenin deposition and spore wall construction.

## Materials and methods

### Plant material and culture conditions

Both Gransden 2004 and *pabB4* lines of *P. patens* (Ashton and Cove, 1977) were examined in this study. *pabB4* was obtained by *N*-methyl-*N*′-nitro-*N*-nitrosoguanidine mutagenesis and shown by conventional genetic analysis through sexual crossing to possess a single *pab* biochemical mutation (Ashton and Cove, 1977; Courtice et al., 1978). This mutation has recently been located in the gene for 4-amino-4-deoxychorismate synthase, which catalyzes the first reaction of the *p*-aminobenzoic acid (PABA) biosynthetic pathway (Prigge et al., 2021). When grown on a medium containing adequate PABA (1.8 and 18 µM for gametophytes and sporophytes, respectively), *pabB4* is phenotypically, i.e., morphologically and developmentally, indistinguishable from the original Gransden wild-type strain from which it was derived. Like the original Gransden wild type, obtained from a single spore isolated from nature by H.L.K. Whitehouse in 1962, *pabB4* produces abundant sporophytes (Ashton and Cove, 1977; Courtice et al., 1978; Singer and Ashton, 2007; Singer et al., 2007) containing numerous viable spores. Spore development and spore coat ornamentation and stratification are identical in Gransden 2004 and *pabB4* lines and are presented together in our observations.

Gametophytes were grown axenically in 30 mL glass tubes containing ABC medium (Knight et al., 1988) solidified with 1.5% agar and supplemented with 1.8 µM PABA. The cultures were maintained under continuous white light (25–40 μmol·m^−2^·s^−1^) at 22–24°C and 30–50% relative humidity. To produce sporophytes, 2–3-month-old gametophytes were subjected to cold stress at 16°C for 3 weeks and then irrigated with water containing 18 µM PABA to facilitate fertilization and support growth and development of the PABA-requiring sporophytes.

### Acetone extraction and acetolysis

Spores from mature orange capsules were suspended in acetone (30 mL) and stirred under reflux for 6 h. The defatted spores were washed with water to give acetone-treated spores. Then, the remaining spores were subjected to acetolysis in freshly prepared 9:1 (by vol.) acetic anhydride and sulfuric acid at 70°C for 20 min. More than 10 sporophytes from three culture tubes were used in each treatment.

### Light microscopy

Whole sporophyte images were obtained using a Nikon Eclipse 80i compound light microscope equipped with a DS-Ri1 digital camera.

Thick sections (1–1.5 µm) of sporophytes processed for transmission electron microscopy (TEM) and in resin blocks were mounted on glass slides and stained with 1.5% toluidine blue in distilled water to monitor the spore stage and integrity using light microscopy. Digital images were captured on a Leica DM5000 B compound microscope.

### Transmission electron microscopy

Capsules of mature sporophytes were punctured with a razor blade or a fine needle to facilitate infiltration of solutions and resin. The specimens were fixed in 2% glutaraldehyde in 50 mM sodium phosphate buffer (pH 7.2) for 1 h at room temperature, then overnight at 20°C. Following three rinses in buffer for 30 min each, the sporophytes were post-fixed for 1 h in buffered 2.5% OsO_4_ followed by three 10-min rinses in distilled water. The plants were dehydrated in a graded ethanol series, rinsed three times in 100% ethanol, and slowly infiltrated over 4–7 days by increasing the ratio of Spurr’s resin to ethanol. The plants were placed in molds with fresh resin and cured for 16 h at 65°C. Thin sections (90–95 nm) were collected on nickel grids and post-stained with methanolic uranyl acetate and basic lead citrate. The specimens were observed on a Hitachi H7650 TEM at 60 kV, and images were captured digitally.

### Scanning electron microscopy

Three to six capsules were harvested from controls and each treatment, air-dried, opened on stubs to disperse the spores, and processed according to Rabbi et al. (2020). Germinated spores were fixed per the TEM protocol and critical point dried rather than being infiltrated with resin. The specimens were sputter-coated with 50 nm of Au/Pd using a Denton Desk II Vacuum Sputter Coater and imaged using a scanning electron microscope (SEM) (QUANTA FEG 450; FEI) with 5 kV acceleration voltage.

## Results

**FIGURE 1:**
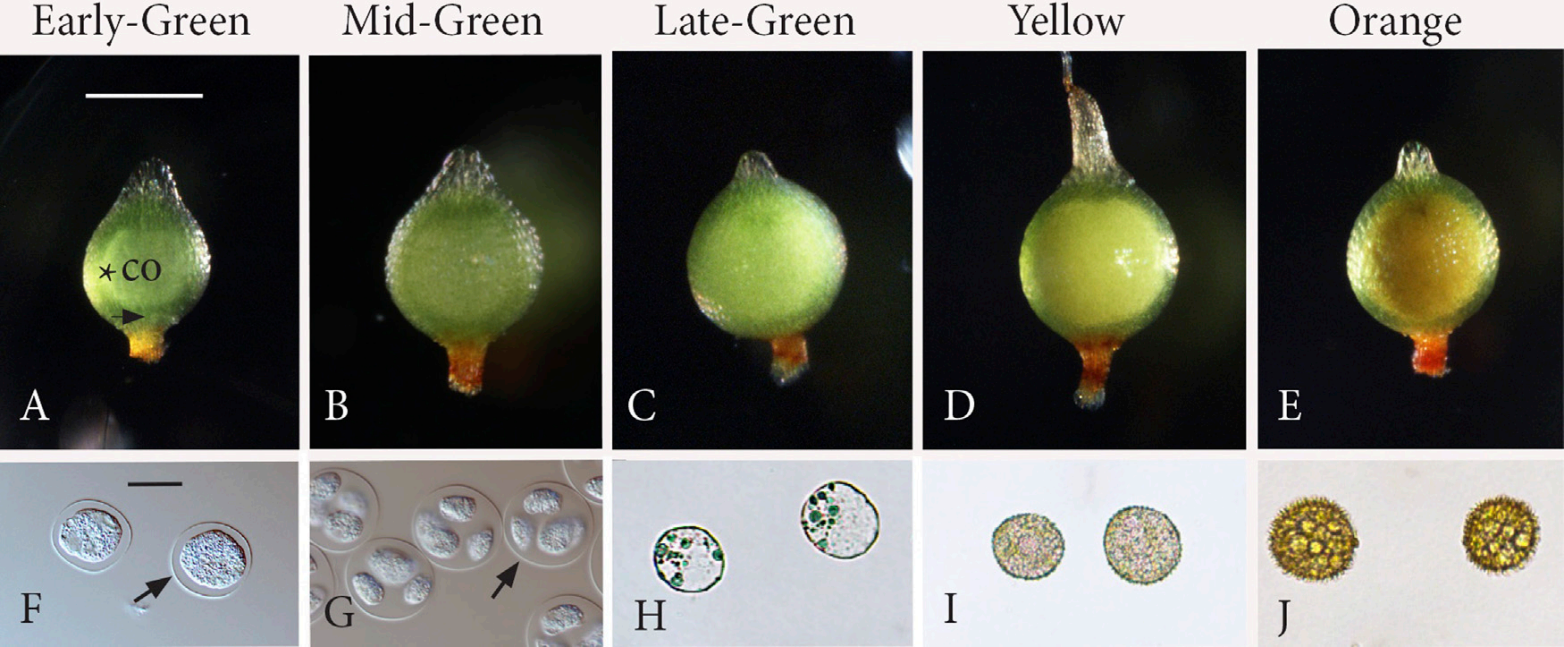
Five stages of sporogenesis. Capsules **(A−E)** and corresponding spore content **(F−J)**. **(A)** Stage 1: *Early-Green*. Green expanding capsule that contains a central columella (co) surrounded by a narrow ring of archesporial tissue (*). Stomata (arrow). **(B)** Stage 2: *Mid-Green*. Following meiosis, the spherical capsule is fully expanded with a larger central mass of tetrads in the locular matrix, obscuring the columella for the remainder of development. **(C)** Stage 3: *Late-Green*. Green capsules with an expanded inner whitish mass of free spores that are greatly enlarged compared with those in tetrads in Figures 1B, G. **(D)** Stage 4: *Yellow*. Spores occupy a yellow mass that fills the center of the capsule. **(E)** Stage 5: *Orange*. Fully developed spores comprise a central orange mass in mature capsules. **(F)** Stage 1: rounded spore mother cells surrounded by the thickened extraprotoplasmic matrix inside the thin spore mother cell (SMC) wall (arrow). **(G)** Stage 2: round tetrads surrounded by the SMC wall (arrow) with nascent spores suspended in the extraprotoplasmic matrix and separated by an intersporal matrix. **(H)** Stage 3: young rounded free spores. **(I)** Stage 4: spores with a developing perine. **(J)** Stage 5: mature spinose spores. Scale bars: **(A−E)** = 500 μm; **(F−J)** = 20 μm.

The size and appearance of capsules at five key stages of sporogenesis (Daku et al., 2016) from SMC to mature spore are illustrated in Figure 1 and described as follows.

*Stage 1: Early-Green* (Figures 1A, F)*:* Rounded SMCs (Figure 1F) are evident in green-expanding capsules, which are oval in shape, have stomata, and contain a cylindrical layer of archesporial tissue surrounding the central columella (Figure 1A). *Stage 2: Mid-Green* (Figures 1B, G)*:* Following meiosis, the spherical capsule is fully expanded with a larger opaque mass (Figure 1B) that contains rounded tetrads with nascent spores (Figure 1G) suspended in a matrix. The columella is obscured at this stage and the following stages due to the expanded spore mass. *Stage 3: Late-Green* (Figures 1C, H)*:* Young rounded free spores (Figure 1H) have enlarged compared to spores in tetrads in stage 1 (Figure 1G) and are found in green capsules with an expanded inner mass that has a white tinge (Figure 1C). *Stage 4: Yellow* (Figures 1D, I)*:* Spores are encased in the developing spore walls (Figure 1I) and comprise a yellow mass that fills the center of the capsule (Figure 1D). *Stage 5: Orange* (Figures 1E, J)*:* Fully developed spores (Figure 1J) comprise a central orange mass in mature capsules (Figure 1E).

**FIGURE 2:**
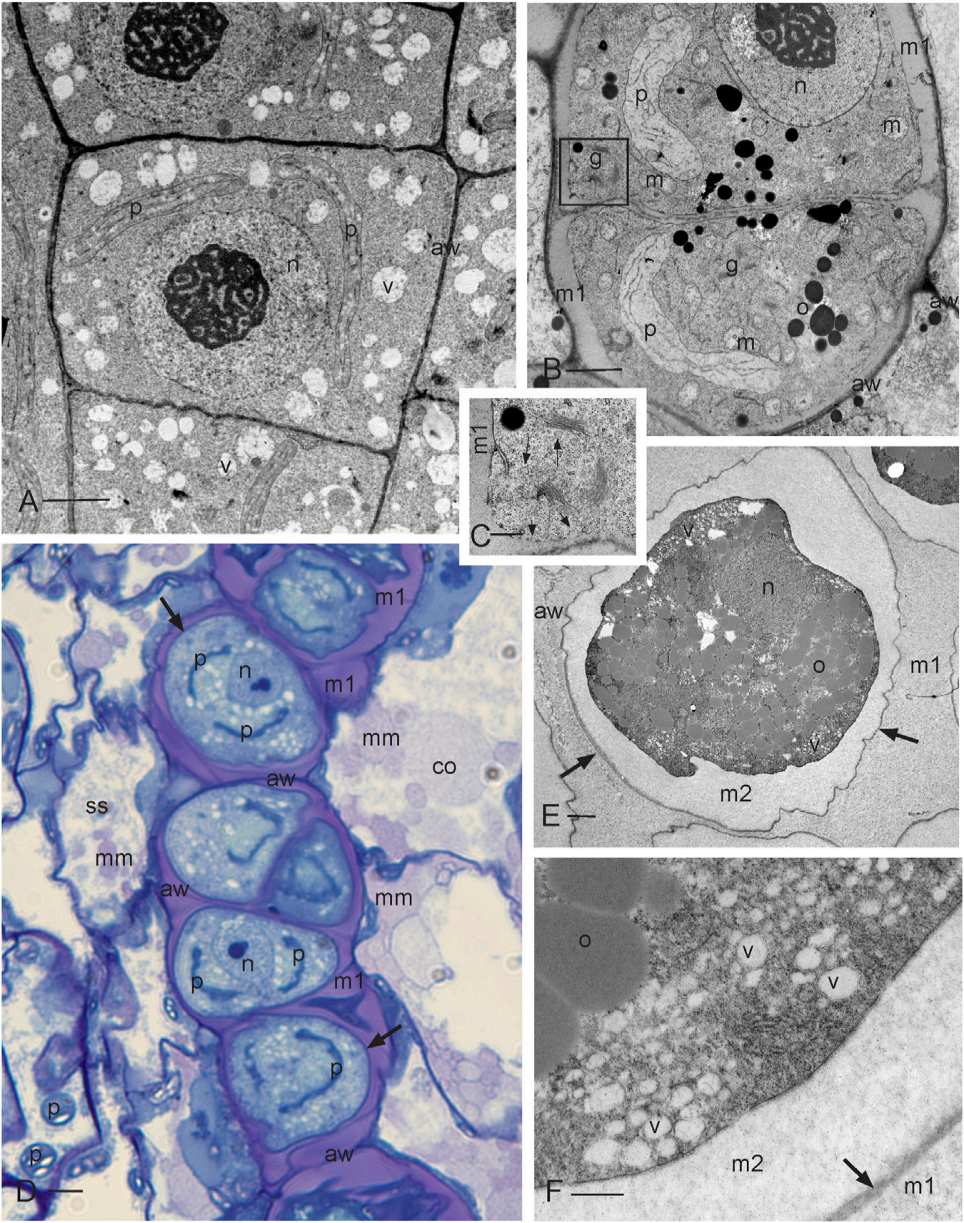
Development of sporogenous tissue. **(A)** Transmission electron microscope (TEM) image of archesporial cells showing thin walls (aw), a dense cytoplasm, and a large central nucleus (n) with prominent nucleolus and scattered vesicles (v). Two plastids (p) are migrating to the opposite poles in preparation for mitosis. **(B)** TEM of differentiating SMCs following the final mitosis. Cells contain a large nucleus (n), a single plastid (p), numerous mitochondria (m), oil droplets (o), and abundant Golgi bodies (g) that deposit cell wall material through exocytosis, forming a matrix (m1) that rounds and separates SMCs from each other and from archesporial cell walls (aw), the inner capsule wall (spore sac) and columella. **(C)** Enlargement of the Golgi apparatus in the box outlined in **(B)** showing Golgi vesicles (long arrows) and vesicles recently fused with the plasmalemma (short arrows) that deposit matrix 1 and rounds SMC protoplasts. **(D)** Light micrograph (LM) of the developing SMC layer in the archesporium surrounded by a spore sac (ss) (inner capsule wall layer) and columella (co). The matrix material (mm) is deposited by the spore sac and columella further contributing to the locular matrix (m1) that surrounds and suspends spores during development. SMCs are delimited by a thin cell wall (arrows) and contain a large nucleus (n) and two plastids (p) that will undergo another round of division to produce four plastids in preparation for meiosis. The original archesporial cell walls (aw) are visible. **(E)** TEM of an SMC that is embedded in the locular matrix (m1) and has deposited a second matrix (m2) between the thin wall (arrows) and the protoplasm. Archesporial walls (aw) are disintegrating. The SMC contains dense cytoplasm, oil droplets (o), nucleus (n), and numerous peripheral vesicles (v). Plastids are not in the section. **(F)** Enlargement of SMC suspended in the locular matrix (m1) showing oil droplets (o) and numerous vesicles (v), containing a faint fibrillar material, depositing the extraprotoplasmic matrix (m2) between the plasmalemma and the thin SMC wall (arrow). Scale bars: **(A, B, E)** = 2.0 μm; **(C, F)** = 0.5 μm; and **(D)** = 5.0 μm.

**FIGURE 3:**
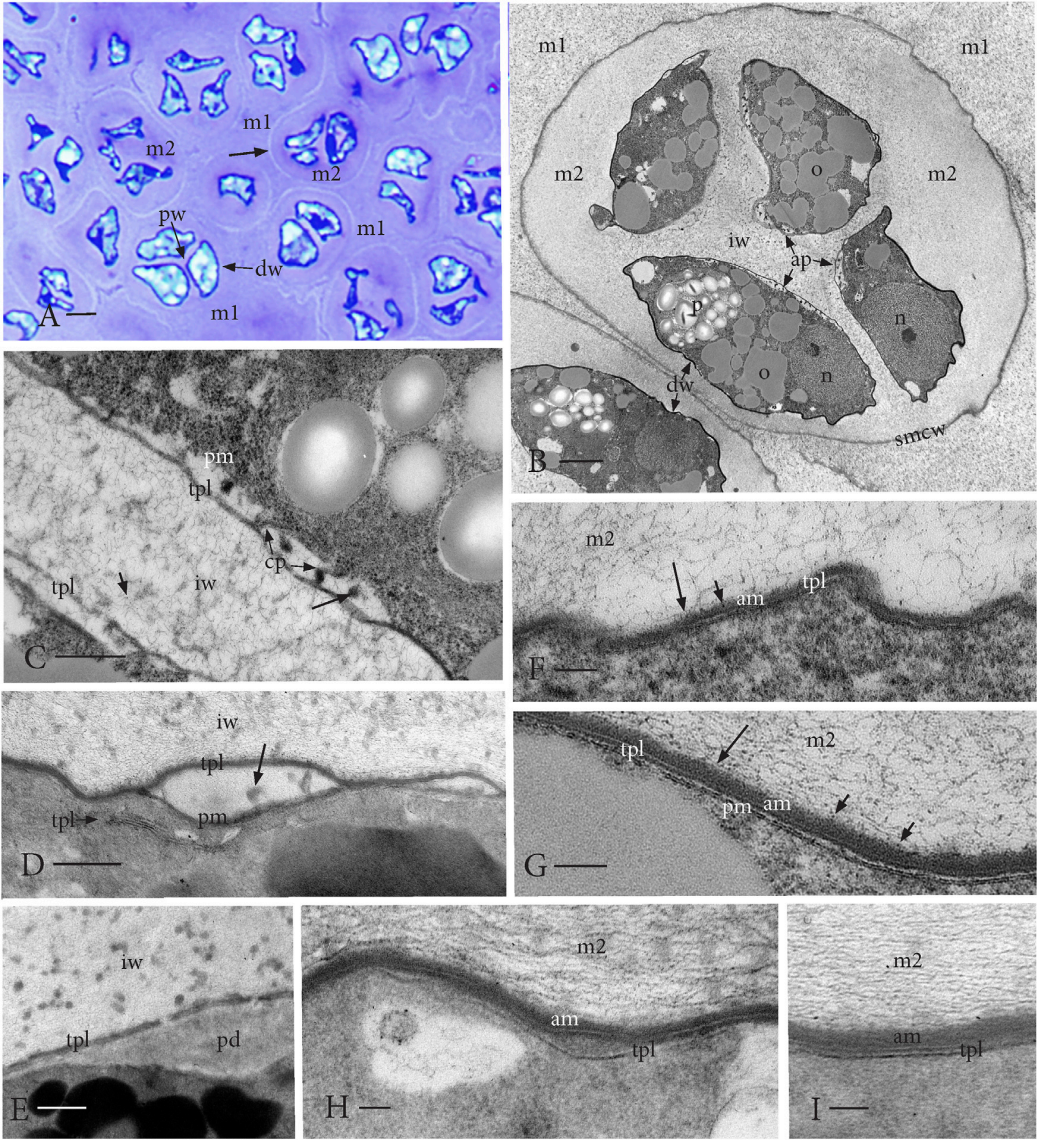
Nascent spores in tetrads in a fully expanded capsule. **(A)** LM of the newly formed tetrads embedded in the locular matrix (m1) derived primarily from the capsule tissue. The extraprotoplasmic matrix (m2) inside a thin SMC wall (arrow) surrounds the distal side (dw) of spores that are wavy in outline. An intersporal matrix separates spores in each tetrad along the proximal spore walls (pw). **(B)** TEM of nascent spores in a tetrad enclosed in the SMC wall (smcw) and embedded in the locular matrix (m1). Aperture (ap) formation is visible along proximal walls of spores where an intersporal matrix/wall (iw) separates spores. Wavy thin distal spore walls (dw) have occasional breaks. Dense spores contain a nucleus (n), starch-filled plastid (p), and abundant oil (o). **(C)** TEM of aperture initiation along the proximal wall in two adjacent spores showing regions of a loose fibrillar material with cytoplasmic projections (cp) and dense amorphous strands (long arrow) between the plasmalemma (pm) and single peripheral tripartite lamella (TPL). The intersporal matrix (iw) contains irregular fibril inclusions and dots (short arrow). **(D)** TEM of layers of TPL in the spore cytoplasm near the developing aperture delimited by a single TPL and plasmalemma (pm) with inclusions (arrow). Fibrillar intersporal wall (iw) contains a scattered dense material. **(E)** TEM of the developing granular pad (pd) in the center of aperture covered by an interrupted TPL layer. Fibrous intersporal wall (iw) with scattered small dense globules. **(F−I)** Initiation and early development of the wavy distal spore wall. **(F, G)** TEMs of single TPL deposited from the spore cytoplasm, while fibrous (long arrow) and dotted (short arrow) material from the extraprotoplasmic matrix surrounding the tetrad (m2) accumulates on the spore outside forming an amorphous layer (am). **(H, I)** Additional TPL from the spore is added to form a two-layered lamella on the distal surface. Amorphous layer (am) thickens, and the extraprotoplasmic matrix (m2) contains faint dense globules. Scale bars: **(A)** = 5.0 μm; **(B)** = 2.0 μm; **(C−E)** = 0.5 μm; and **(F−I)** = 0.1 μm.

**FIGURE 4:**
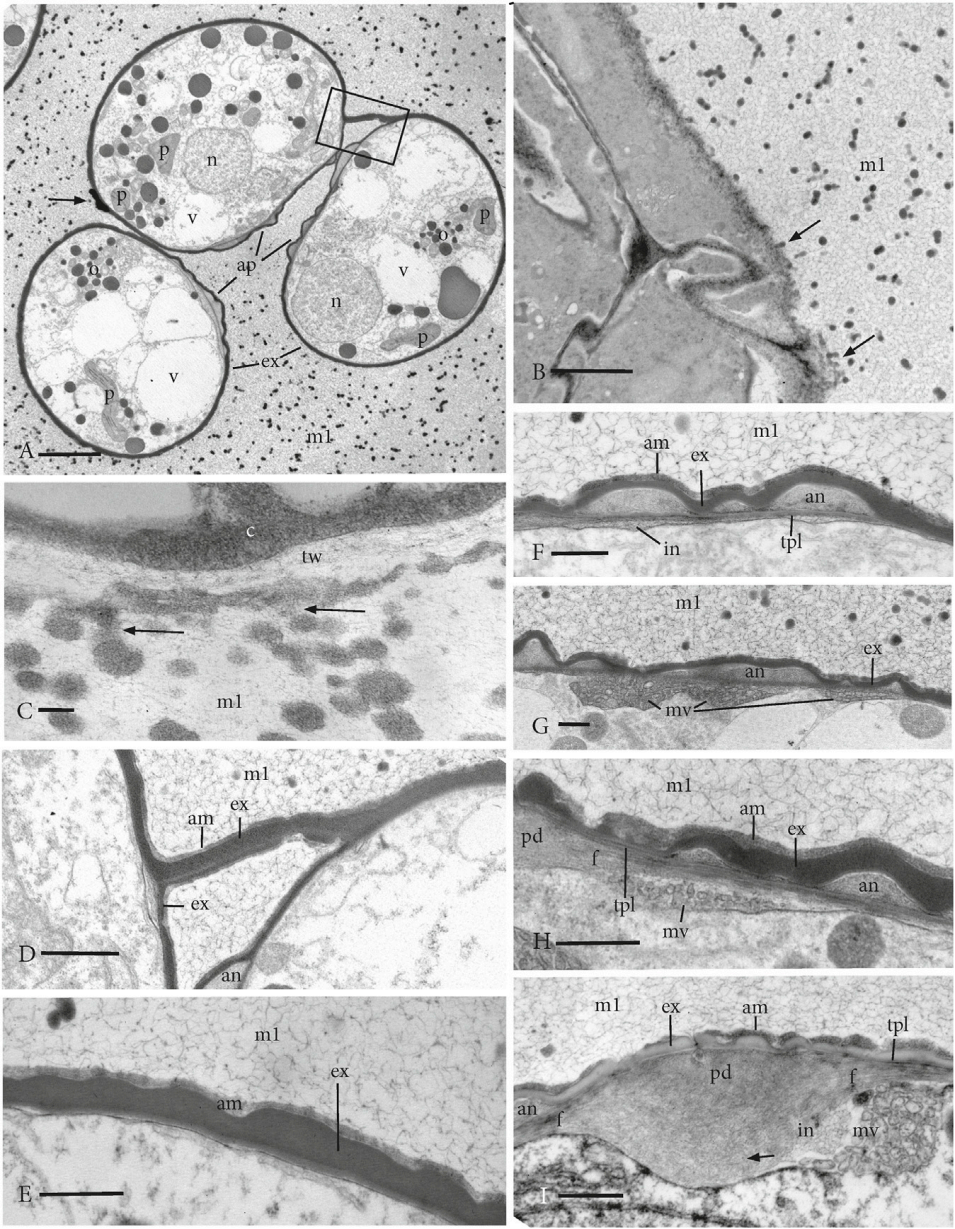
TEMs of young expanded free spores surrounded by sporopollenin globules distributed throughout the locule matrix (m1) that has merged with matrix 2 and the intersporal matrices. **(A)** Three spores in a tetrad that contain sparse cytoplasm with a nucleus (n), plastids (p), scattered oil droplets (o), and numerous vacuoles (v). The exine (ex) delimits the spore, and the apertures (ap) extend along the proximal surface of each spore. Apertures are sectioned only along the annulus region, and no central pad is visible. The arrow denotes the remnant of the SMC wall, and the box outlines a portion of the SMC wall that connects adjacent spores. **(B)** Tapetal cells of the inner capsule wall contain dense cytoplasm and are coated with materials (arrows) similar to globules in the locule matrix (m1). **(C)** Higher magnification showing a dense cytoplasm (c) in the tapetal cell and electron-lucent tapetal cell wall (tw) with the accumulation of a coating of dense material and attached globules (arrows) that are also dispersed in the locule matrix (m1). **(D)** Enlargement of the area in box in **(A)** showing adjacent spores connected by the remnant SMC wall with exine (e) and amorphous zone (am), suggesting both layers are composed of extrasporal materials. an, annulus and m1, locule matrix. **(E)** Distal spore surface showing homogeneous exine (e) with a wavy outer boundary, covered by an amorphous layer (am) and surrounded by a fibrillar network of the locule matrix (m1) with sporopollenin globules. **(F−I)** Developing aperture on the proximal surface surrounded by a fibrillar network of the locule matrix (m1) with sporopollenin globules. **(F)** A foundation layer of 1–3 TPL underlies the annulus (an) that is covered by an undulating exine (ex) and an amorphous layer (am). The intine (in) begins development in patches under the aperture but is not yet visible under the distal exine. **(G)** Multivesicular bodies (mv) contribute to the developing intine under the annulus (an) that is covered by an undulating exine (ex). **(H)** Junction between the annulus (an) and the central pad (pd) of the aperture that is overarched by an undulating exine (ex) and an amorphous layer (am). TPL underlie the exine and subtending fibers run from the pad beneath the annulus. Intine development involves aggregates of vesicles in multivesicular bodies (mv). **(I)** Central pad (pd) of the aperture has fibers (f) that extend beneath TPL and annulus (an). Intine (in) development is extensive under the pad and involves aggregates of vesicles in multivesicular bodies (mv). Remnants of vesicles (arrow) are faint under the pad. The irregular exine (ex) is sparsely covered by an interrupted amorphous layer (am). Scale bars: **(A)** = 5.0 μm; **(B)** = 2.0 μm; **(C)** = 0.2 μm; **(D)** = 1.0 μm; and **(*E*–I)** = 0.5 μm.

**FIGURE 5:**
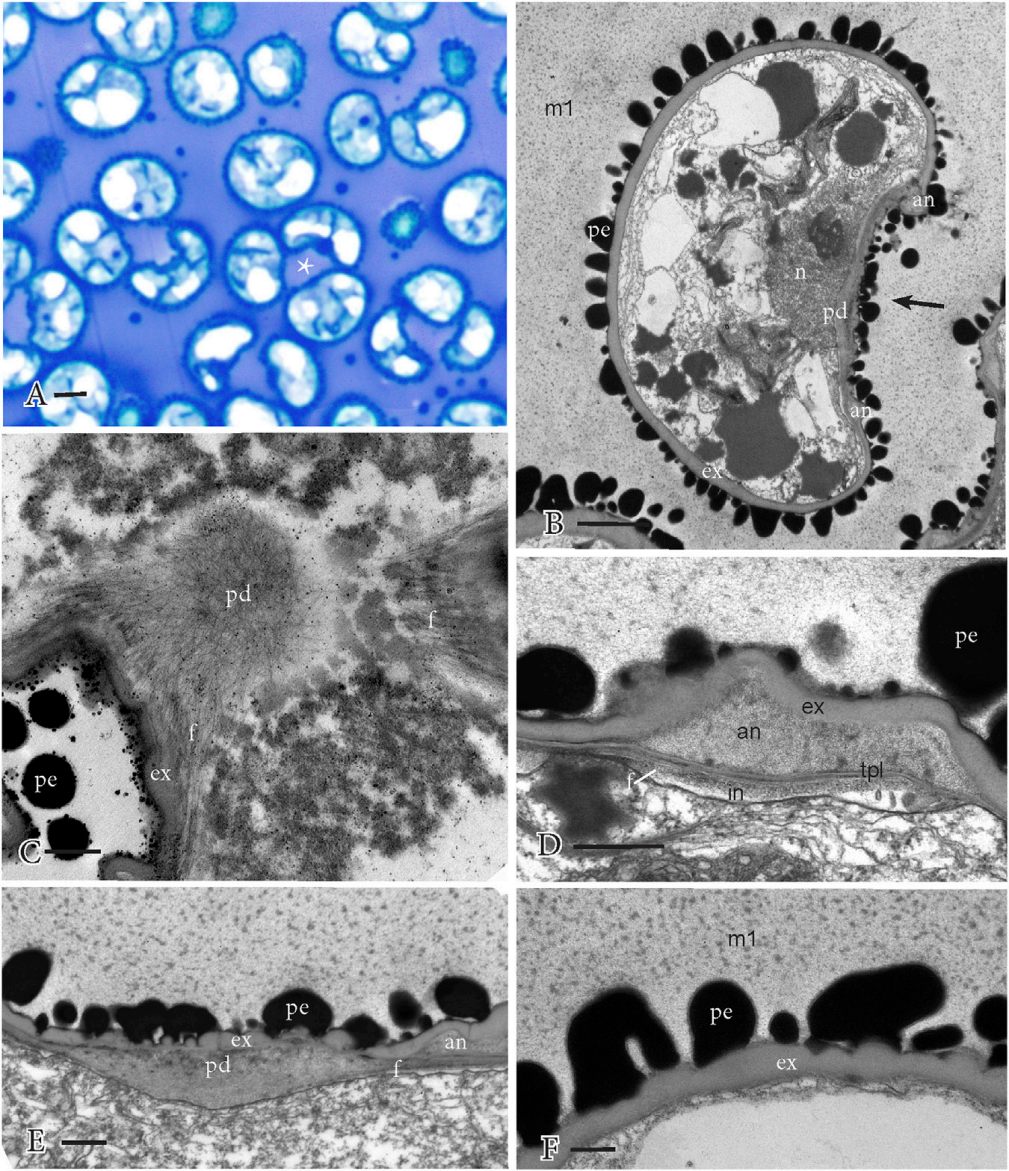
Perine development and aperture substructure. Perine ornamentation formed through the adherence of sporopollenin globules from the previous stage. The locular matrix (m1) is no longer fibrillar and contains abundant small dense inclusions. **(A)** LM of spores dispersed in the locule in tetrahedral arrangement and often with a concave proximal spore surface (*). **(B)** TEM of spore with a sparse cytoplasm, surrounded by the exine (ex) and truncated perine (pe) spikes along the distal wall. The aperture extends along the proximal face (arrow), which is concave and covered by an irregular perine. The aperture pad (pd) and the annulus (an) are reduced in stature. The globules are absent from the locular matrix, and only small dense inclusions remain. **(C)** TEM surface section of the aperture showing a fibrillar network of the central pad (pd) and radiating fibers (f). (ex), exine; (pe), perine. **(D)** TEM details of the annulus (an) regions of the aperture, which is inserted between the TPL and undulating exine (ex). Fibers (f) extend from the pad under the TPL and intine (in) is expanding inwardly. An irregular perine (pe) covers the spore. **(E)** TEM details of the central pad (pd) region of the aperture on the proximal side of a concave spore shows the fibers (f) that extend beneath the annulus (an) and the irregular exine (ex) and perine (pe). Small dense inclusions are dispersed in the matrix around the spore. **(F)** TEM details of the distal spore side with exine (ex), truncated perine (pe) ornamentation, and no intine development. Small dense inclusions are dispersed in the matrix (m1) around the spore. Scale bars: **(A)** = 5.0 μm; **(B)** = 2.0 μm; and **(C−F)** = 0.5 μm.

**FIGURE 6:**
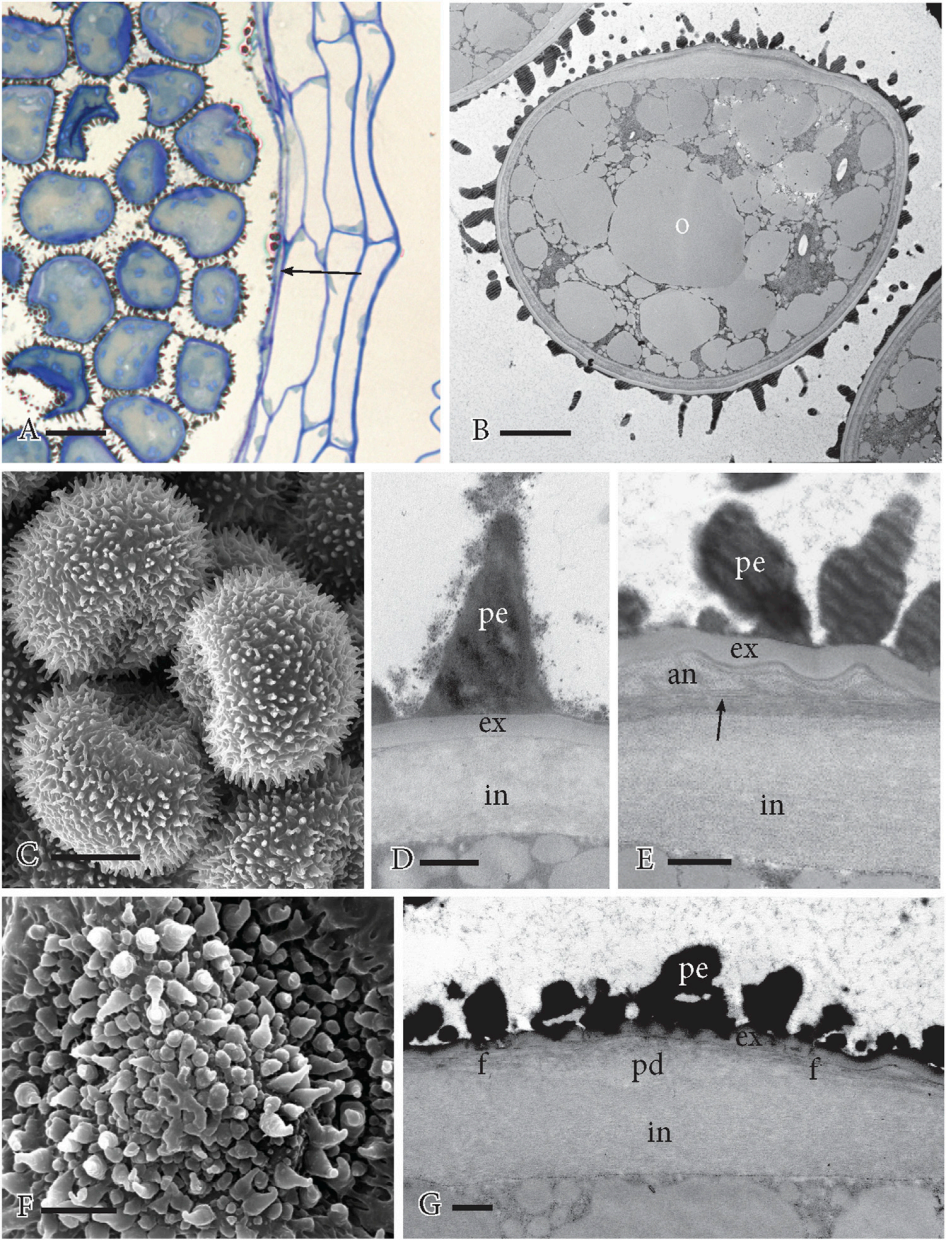
Mature spores. **(A)** LM section showing spiny spores filling the capsule locule and the 4–5-layered capsule wall. The inner capsule wall (arrow) is shrunken with adhering sporopollenin. **(B)** TEM of the mature spore engorged with oil (o) with organelles between. The proximal aperture faces up, and the distal spore is covered in perine spines. **(C)** SEM of three spores of a tetrad. The proximal side is concave, and the distal side is covered in spines. **(D)** TEM of the distal spore wall showing perine spine (pe), evenly thickened exine (ex), and thick inner intine (in). **(E)** TEM of the aperture region at the annulus. Wall layering from outside to inside is perine (pe), wavy exine (ex), annulus (an), TPL (arrow) and subtending fibers, and thick intine (in). **(F)** SEM of the aperture on the proximal side of the spore with irregular perine ornamentation. The central pad subtends the projecting mid-region of the aperture. **(G)** TEM of the central pad region of the aperture. The perine (pe) and exine (ex) are irregular and thin, fibers (f) radiate on all sides from the pad (pd), and the intine (in) is thickened. Scale bars: **(A)** = 20 μm; **(B)** = 5.0 μm; **(C)** = 10 μm; **(F)** = 2.5 μm; and **(D, E, G)** = 0.5 μm.

**FIGURE 7:**
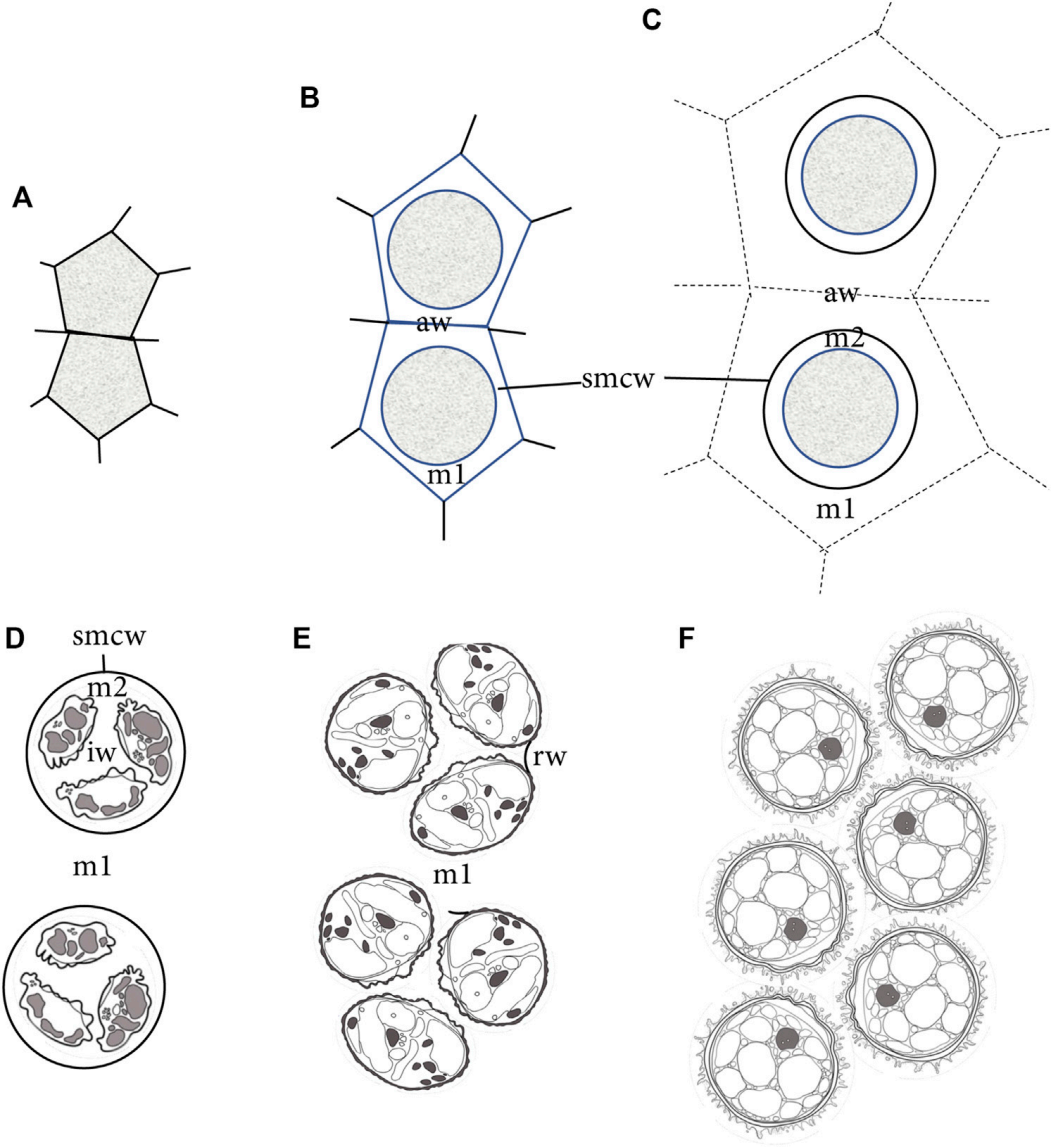
Diagrammatic representation of sporogenesis in *Physcomitrium patens,* illustrating the sequence of development of wall/matrices relative to the sizes of SMCs and spores. **(A)** Two newly formed angular SMCs surrounded by archesporial cell walls. **(B)** Deposition of matrix (m1) material from the SMC rounds the protoplasm and separates SMCs from archesporial cell walls (aw). A thin cell wall (smcw) is deposited around the SMCs. **(C)** SMCs deposit an extraprotoplasmic matrix (m2) between the thin SMC wall (smcw) and protoplasm. Further deposition from tapetal cells adds to the locular matrix (m1), increasing the distance between SMCs and obscuring archesporial cell walls (aw). **(D)** Nascent tetrads are suspended in the locular matrix (m1), and each contains four small spores separated by a newly made intersporal wall/matrix (iw), embedded in the extraprotoplasmic matrix (m2) and surrounded by an SMC wall (smcw). **(E)** Rounded free spores are embedded in the locular matrix (m1) and have expanded beyond the SMC wall, leaving random remnant wall connections (rw) between the sister spores in a tetrad. Apertures are visible along proximal surfaces where spores in tetrads face each other. **(F)** Expanded mature spores fill the capsule locule.

**FIGURE 8:**
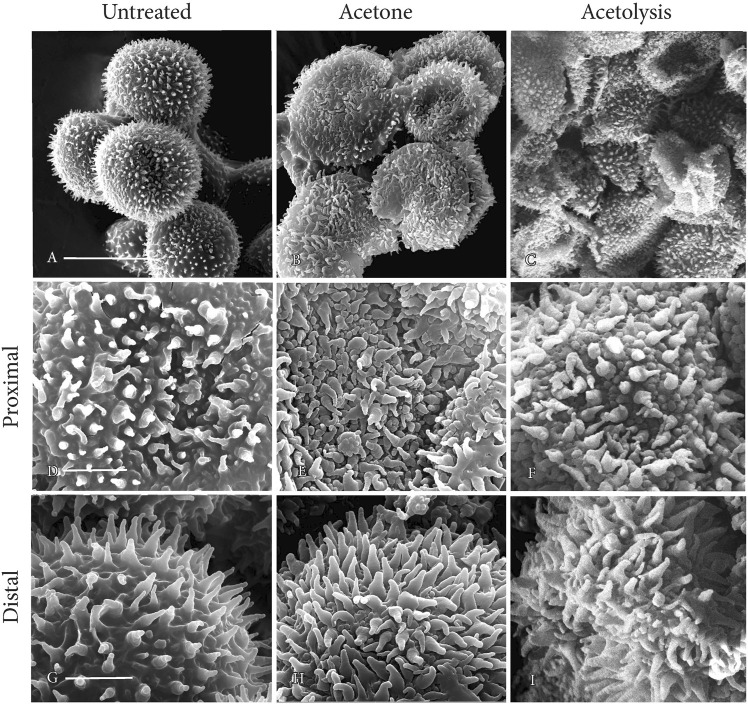
SEMs of mature spores. Perine ornamentation remains intact on both proximal and distal sides of spores after acetone treatment and acetolysis. **(A, D, G)** untreated; **(B, E, H)** acetone treated; and **(C, F, I)** acetolysis treated. Scale bars: **(A−C)** = 20 μm; and **(D−I)** = 5.0 μm.

**FIGURE 9:**
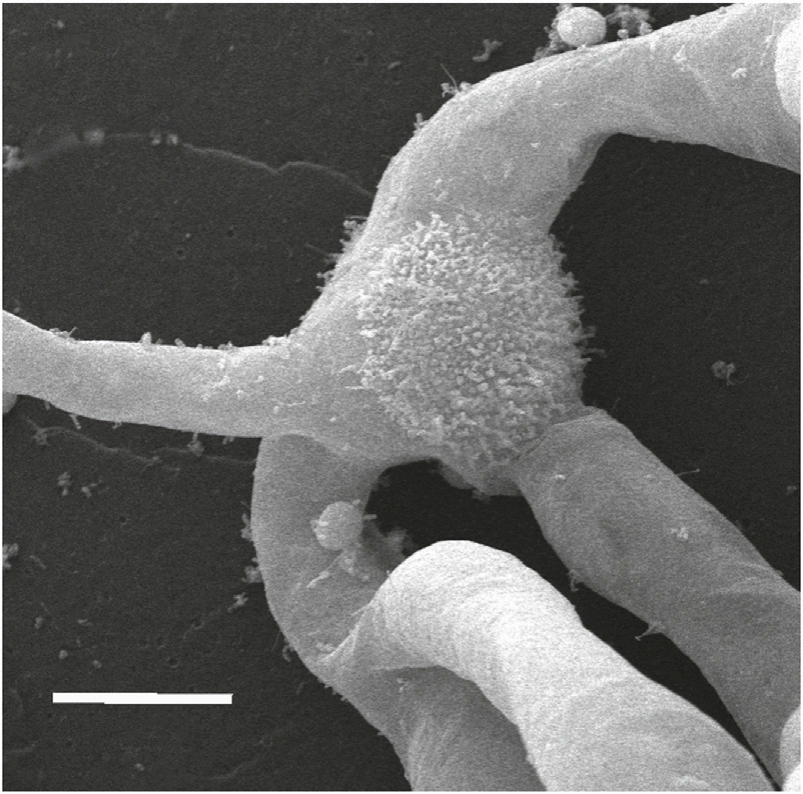
SEM of the germinated spore showing the intact distal spore wall with three protonemata emerging from the proximal side of the spore. Scale bar = 20 μm.

Ultrastructural details of the five stages of sporogenesis in Figure 1 are described as follows with supporting images (Figures 2–6). Spore wall development is followed in the two morphologically distinct areas of the spore wall: the proximal wall, where spores in each tetrad face each other and where the aperture forms, and the distal wall, which is directed toward the SMC wall. Figure 7 presents a diagrammatic illustration of the sequence of deposition of cell walls and matrices as they correspond to changes in size and to the relationship of SMCs and spores during sporogenesis. Acetone and acetolysis treatments are illustrated in Figure 8 and a germinated spore in Figure 9.

### Stage 1: Archesporial cells and spore mother cells

Developing archesporial cells are angular and thin-walled and contain dense cytoplasm with scattered vacuoles and a central nucleus containing a prominent nucleolus (Figure 2A; Figure 7A). Each cell contains a single elongated plastid that divides prior to mitosis, serves as the focal point for mitotic spindles, and ensures that one plastid is distributed into each of the two daughter cells. Archesporial tissue comprises a narrow and distinctive cell layer sandwiched between the large columella cells and inner capsule wall or spore sac (Figure 1A; Figure 2D). Prior to meiosis, the monoplastidic SMCs contain numerous mitochondria, scattered oil droplets, and Golgi bodies (Figures 2B, C). They begin to lay down a matrix (matrix 1) *via* Golgi exocytosis that separates the original archesporial cell walls from the protoplasm and rounds out the differentiating SMCs (Figures 2B, C; Figure 7B). The cells of the capsule wall layers contain chloroplasts that impart green color to the capsule (Figure 1A). The inner capsule wall (spore sac) and columella continue to deposit matrix 1, separating SMCs in an extensive locular matrix (Figure 2D; Figure 7C). At this time, the round SMCs lay down a thin wall, followed by a wider extraprotoplasmic matrix (matrix 2) between the plasmalemma and thin original SMC wall (Figure 1F; Figures 2E, F; Figure 7C). This second matrix thickens *via* the exocytosis of large vesicles containing a faintly fibrillar material (Figure 2F). This process occurs before the capsule is fully expanded (Figure 1A). At this stage, however, cell divisions in other capsule tissues are complete and the capsule wall is four to five cells in thickness (not shown), stomata are formed, and the columella is expansive in the capsule center (Figure 1A). Directly prior to meiosis, round SMCs are suspended in a series of matrices/walls that include the original archesporial cell walls, a matrix in which SMCs are embedded (matrix 1), the thin SMC wall, and a matrix that surrounds the SMC protoplasm (matrix 2 or extraprotoplasmic matrix) (Figures 2D–F; Figure 7C). These extracellular matrices are produced in sequence from the structural components of the capsule and are diploid in origin.

### Stage 2: Tetrads with nascent spores encased in SMC walls

This stage is characterized by the initiation of the aperture and the distal spore wall. Capsule expansion is completed following meiosis, and the majority of the locular space is occupied by extracellular matrices with interspersed tetrads (Figure 1B; Figures 3A, B; Figure 7D). During cytokinesis, a wide intersporal wall/matrix is deposited that separates the spores in each tetrad at their proximal faces (Figures 3A, B; Figure 7D). This matrix is fibrillar with irregular dense inclusions and minute dark dots (Figures 3B–E). The nascent spores are wavy in outline and contain a large starch-filled plastid, abundant oil droplets, and an off-center nucleus (Figure 3B). The tetrads are encased in a well-delineated SMC wall and the spores are embedded in both the extraprotoplasmic matrix (matrix 2) contributed by the SMC and the intersporal wall or matrix (Figure 1G; Figures 3A, B). Aperture development begins with the deposition of a single tripartite lamella (TPL) (Figures 3C, D). The plasmalemma and TPL separate from each other in localized regions along the aperture where a fibrillar material is deposited (Figure 3C). Both cytoplasmic projections and dense amorphous strands are visible between the single TPL and spore protoplasm (Figure 3C). Additional layers of TPL are produced in the spore cytoplasm and are deposited in the developing aperture (Figure 3D). A granular pad originates at the center of the aperture and is covered by a single disrupted TPL (Figure 3E). Concomitant with the initiation and development of the aperture, the distal spore wall initiates development with the production of a single TPL around the wavy spore periphery (Figure 3F). As spore expansion occurs, the spore swells in a distal direction and the external spore surface accumulates an amorphous layer of materials (fibers, dots, and dense inclusions) likely derived from the matrix (Figures 3F, G). The amorphous coating on the distal spore reaches and maintains a thickness of approximately 50 nm. A second layer of TPL assembles in the spore cytoplasm and is contributed to the spore wall (Figures 3H, I). This two-layered TPL layer serves as the foundation for sporopollenin deposition in exine formation.

### Stage 3: Free spores with aperture and exine development

From nascent spores in tetrads in stage 2 (Figure 1B) to free spores (Figure 1C), the spores expand in all directions, especially in width, and begin to fill the locular and intersporal spaces (Figure 4A). The average spore width increases by nearly 50% and the length on average increases 7%. As this occurs, the wavy distal wall is drawn taut and the spores become more rounded (Figure 4A). In addition to the fibrillar network and small dots found in previous stages, the locular matrix (matrix 1) and intersporal matrix now contain evenly distributed dense globules, between 50 and 250 nm in diameter (Figure 4). The shrinking cells of the inner capsule wall and columella contain dense cytoplasm and are partially covered by a material that is similar in density to and attached to the globules in the matrix (Figures 4B, C). Spore expansion occurs in synchrony with exine development around the spore (Figures 4D–I) and elaboration of the aperture on the proximal face (Figures 4F–I).

In the free spore stage, the extraprotoplasmic matrix (matrix 2) no longer exists because the spores have expanded beyond the confines of the SMC wall, thereby “freeing” spores from tetrads (Figure 7E). A distinct exine layer surrounds the spore and obscures the TPL foundation layer (Figures 4D–I). The exine boundary is straight on the inner surface and wavy on the outer surface where it is covered by a less dense amorphous layer (Figure 4E). Remnant SMC wall connects adjacent spores until this connection is severed by spore expansion and spores are freed from each other (Figure 4A *;* Figure 7E). Following the deposition of the foundation layer and its impregnation with sporopollenin contributed from the spore, the exine layer thickens centrifugally on the outside of the spore. An extrasporal contribution to the exine is corroborated by the accumulation of exine covered with an amorphous layer along the connecting SMC walls that are not directly associated with any spore cytoplasm (Figure 4D).

The aperture in free spores consists of a thin exine that undulates over the surface and is covered by an interrupted amorphous layer of materials presumably derived from the intersporal matrix (Figures 4F–I). The aperture consists of a swollen central pad that is surrounded by a more elaborate annulus, which is visible in section as protruding regions of finely granular gray material (Figures 4F–I). The annulus derives from the development of the zones visible in Figures 3C, 3D and forms a broad ring around the central pad along the remaining proximal spore surface (Figures 4F–I). A foundation layer of 1–3 TPL underlies the annulus protrusions and extends above the central pad beneath the exine (Figures 4F, H, I). The central pad is covered by a thin irregular exine with scattered amorphous material (Figure 4I). Splayed fibers radiate from the central pad below the foundation layer (Figures 4H, I). Intine development begins at this stage along the aperture but is delayed along the distal wall layers (Figures 4F–I). Intine is deposited irregularly beneath the foundation layer of the annulus and involves aggregates of vesicles containing dark dots (Figures 4G, H). Intine deposition beneath the pad is particularly extensive and involves massive multivesicular units beneath the splayed fibers (Figure 4I). Faint outlines of similar vesicles are visible in the intine as it widens in the region that subtends the pad.

### Stage 4: Perine production

During perine development, the free spores are expanded to their final sizes and often exhibit concave proximal faces (Figures 5A, B). The spores may be associated in tetrads because they are suspended in the matrix and not because they are directly connected (Figures 5A, B). This stage of development is characterized by the progressive accumulation of sporopollenin globules from the locule on the surface of the exine, resulting in a controlled development of the spiny surface ornamentation. The spines are constructed on the peaks of the wavy outer exine surface visible in the previous stage. As spores expand, the large sporopollenin globules of stage 3 (Figure 4) adhere to the spore surface and disappear from the locular matrix, resulting in truncated undeveloped perine spines surrounded by small dense inclusions (Figure 5B). On the proximal side, the aperture is fully developed and consists of a central pad comprising a fibrous network with radiating fibers underlying the annulus (Figures 5C, D). One or two TPL are visible between the annulus and the fibers that radiate from the pad (Figure 5D). The ornamentation on the surface of the aperture (Figures 5B, D, E) is less regular and poorly developed compared to that on the distal spore surface (Figures 5B, F). The exine undulates, especially, over the annulus (Figures 5D, E). Intine development continues under the aperture (Figure 5D) but is not visible under the exine on the distal spore region (Figure 5F).

### Stage 5: Mature spores

Mature spores fill the locule, contain abundant oil with interspersed organelles, and are surrounded by perine spines (Figure 7F). The capsule wall is clearly four- or five-layered with the inner capsule wall cells (spore sac) collapsed, devoid of content, and sparsely covered with sporopollenin aggregates (cf, orbicules) (Figure 6A). Elongated spines of equal length decorate the distal wall surface (Figures 6B–D; Figure 7F). A thick intine comprises the innermost spore wall that extends around the entire spore (Figures 6B, D, E, G). The proximal surface may be convex (Figure 6B) or concave (Figure 6C), reflecting the flexibility of the underlying aperture. The central aperture pad is obscured by the thickened intine but the fibers that radiate from it are often visible under the annulus (Figure 6G) as are the TPL between the fibers and annulus (Figure 6E). Exine undulation is visible over the annulus (Figure 6E). In comparison to the distal ornamentation (Figures 6C, D), the sculptured ornamentation on the aperture is irregular as is the exine (Figure 6E), especially over the central pad (Figures 6F, G).

### Acetone and acetolysis treatments

Compared with untreated spores (Figure 8A), spores after acetone treatment (Figure 8B) and acetolysis (Figure 8C) collapsed under the conditions for SEM because of the absence of cytoplasm. The structural integrity of surface architecture on both proximal and distal spore surfaces remains unaffected following acetone treatment (Figures 8E, H) and acetolysis (Figures 8F, I), compared with controls (Figures 8D, G).

### Germination

The germination of spores occurs at the site of the aperture with as many as four apical cells giving rise to protonemal filaments sequentially rather than simultaneously (Figure 9). The distal spore surface remains intact.

## Discussion

Compared with other bryopsid mosses, the capsule of *P. patens* is highly reduced. There is no peristome and the capsule wall is only four or five cells thick with a swollen basal region containing few stomata and poorly developed intercellular spaces (Merced and Renzaglia, 2017). Dehiscence is irregular along the capsule in this cleistocarpic moss. The inner capsule wall surrounds the locule in which the spores develop, functioning as the outer spore sac. Together with the columella, these cell layers form the so-called tapetum that often consists of well-defined inner and outer cell layers that surround the sporogenous tissue in mosses with more elaborate capsules (Crum, 2001). These specialized morphological features are important to consider when examining the ontogeny of spores in *P. patens.*

Our detailed ultrastructural study has revealed a coordinated and regimented process during sporogenesis in *P. patens* that involves contributions from diploid capsule tissues and the haploid spore. The process begins in concert with capsule expansion and involves the secretion and assembly of locular materials (matrix 1) that separate and suspend SMCs prior to meiosis. As spores develop, they expand to eventually fill the locular space as they progressively and systematically build their walls. During this developmental process, spores contribute key elements to the spore wall, including the TPL upon which the exine is constructed, the intine, and the elements of the spore aperture, namely, the annulus, the central pad, and the attached radiating fibers. Spores not only undergo dramatic changes in size and in their protective spore wall but also they completely transform their content from nascent spores with minimal cytoplasm to mature spores filled with oils. Ultrastructural features and changes in capsule cells, especially the inner capsule wall and columella, point to the involvement of these cells in all phases of sporogenesis and most likely in capsule dehiscence for spore dispersal. In addition to the embryo with the placental zone of exchange between the dependent sporophyte and nourishing gametophyte (Henry and Renzaglia, 2021), the process of sporogenesis provides a second illustration of the importance of precise collaboration between different generations. In sporogenesis, the diploid capsular tissues and developing spores work in concert to regulate proper spore maturation. This interplay between generations is essential for the development of the highly specialized single-celled spores that serve as perennating and dispersal agents in the transition to a persistent and independent haploid gametophyte.

During early capsule expansion in *P patens*, sporogenous cells are isolated from other capsule cells *via* the deposition of three unique cell walls or matrices as illustrated in Figure 7. The first is an extensive locular matrix (m1) that surrounds and separates SMCs from each other and from capsule cells, and obscures the original angular archesporial walls. Each resulting rounded SMC lays down a second thin wall, followed by a sparsely granular matrix (matrix 2) within which meiosis occurs. During spore differentiation, matrix 1 fills with a network of fibrous material in which sporopollenin globules assemble and are added to the developing spore wall.

As in other mosses and hornworts, meiosis is monoplastidic (Brown and Lemmon, 1990). The single plastid undergoes two rounds of division and one starch-filled plastid is distributed into each spore. Unlike mosses and hornworts in which meiosis is universally monoplastidic, the vast majority of liverworts undergo polyplastidic meiosis with the sporadic occurrence of monoplastidic meiosis in two disparate lineages: *Haplomitrium*, the sister taxon to other liverworts, and scattered complex thalloid taxa (Renzaglia et al., 1994; Shimamura et al., 2003; Brown and Lemmon, 2013).

### Aperture

The polarity of spores is prominent throughout sporogenesis in *P. patens* and is apparent in the production of a structurally distinct distal wall and a proximal wall where the aperture is constructed. As in other mosses, the aperture is trilaesurate and extends across the proximal spore surface along the contact faces of the four spores in the tetrad (Brown and Lemmon, 1981; Gambardella et al., 1994; Brown et al., 2015). The moss aperture is complex and in *P. patens* includes a modified irregular perine, thin interrupted exine, highly thickened intine, and a specialized central disc-like pad that contains callose (Schuette et al., 2009). Fibers radiate from the central pad beneath the foundation layer and the annulus, which consists of wall protrusions that encircle the pad and are inserted between the exine and the intine. This complex architecture is also evident in *Takakia, Oedipodium,* and peristomate mosses, indicating that this spore feature is widespread and evolved early in moss diversification (Brown and Lemmon, 1981; Renzaglia et al., 1997; Brown et al., 2015). Apertures are found in most hornworts and are proximal in location as in mosses, but they are much simpler in design. The modified spore wall of hornwort apertures does not extend across the entire proximal wall but is restricted to the trilete mark and equatorial girdle, and is an area of expanded intine and modified exine with limited sporopollenin deposition. Consequently, upon germination, the hornwort spore is ruptured at the aperture and along the equator, often forming three detachable valves (Renzaglia, 1978; Renzaglia et al., 2009). In contrast, the apertures of pteridophytes and seed plants contain expanded and modified exines and not intines (Tryon and Lugardon, 1991). Pollen apertures are generally thin areas through which the tube emerges but they are typically located distally, although they may occur on any side of the pollen surface. Aperture loss is documented in spores and pollen and has occurred across plant groups. Notable examples are *Andreaea* among mosses and *Dendroceros* among hornworts. Inaperturate pollen grains are found in less-specialized angiosperms, and highly specialized apertures, such as the dicotyledonous colpate pollen type, subsequently evolved in more derived groups (Severova et al., 2022).

In both mosses and hornworts, the aperture is viewed as germinal because it is a weak site in the spore coat through which the elongating protonemata can easily rupture (Reighard, 1967; Olesen and Mogenson, 1978; Brown and Lemmon, 1980). In *P. patens,* as many as four apical cells giving rise to protonemal filaments may emerge through the aperture and usually do so sequentially rather than simultaneously. The complex structure of the moss aperture in *P. patens* and across mosses may reflect functions in addition to germination. One possible additional role is in water imbibition that precedes spore rupture. The apertures of both mosses and hornworts contain much less sporopollenin in irregular exine layers, which would facilitate water intake. The occurrence of a specialized disc-like aperture pad in the *P. patens* spore wall and the exclusive localization of the hydrophilic polysaccharide callose to this pad speak to a likely role of the aperture in imbibition (Schuette et al., 2009).

Based on the observations presented herein, we propose a novel role for the moss aperture and that is in desiccation tolerance. It is clear from our microscopic observations in the LM, TEM, and SEM that the proximal face often becomes concave during development as the spores mature. Upon release, the spores may be rounded or concave. Architectural features of the aperture in both states indicate that the proximal spore surface has the capacity to collapse, forming a concave surface, or expand, forming a rounded configuration. The undulating exine over the aperture is flexible and is able to accommodate changes in spore shape without rupturing the wall. The elaborate construction of the aperture extends across the proximal face and includes a radiating fibrous network that connects the central disc-shaped pad containing callose to the expanded aperture intine. During aperture development, microtubules presumably guide the fibers as they are deposited (Brown and Lemmon, 1981). This organization is reminiscent of the torus and margo structure in aspirating circular-bordered pits of gymnosperms (Hacke et al., 2004). In contrast to the circular-bordered pits that are functional in dead cells and are not able to move once aspirated, the moss aperture appears to be elastic, changing positions as the spore loses and gains water during development and in response to moisture changes in the environment. This elasticity would maintain the integrity of the connection between the spore wall and plasmalemma, thus preventing lethal plasmolysis. Mature spores are engorged with oil and following the release from the capsule have the capacity to remain in a dehydrated state pending favorable conditions for germination. The elasticity of the aperture along the proximal surface would enhance the ability of spores to respond to moisture changes and tolerate extended periods of desiccation. We propose that collapsed spores represent a normal desiccation-tolerant state and are not artefacts of preparatory procedures for microscopy. With a limited amount of sporopollenin in the aperture and equatorial girdle, hornwort spores would similarly have the ability to fold and expand with water availability. Pollen apertures are also designed to fold as water is lost and serve as the primary elements of harmomegathy (folding of pollen grains due to water loss) since they are more elastic than the remainder of the pollen wall (Volkova et al., 2013; Božič and Siber, 2020).

### Exine and sporopollenin

The exine (exospore) is the only sporopollenin-containing wall layer universally present in spores and pollen across land plants (Blackmore et al., 2007). Sporopollenin is a heteropolymer composed of polyhydroxylated polyketides, hydroxylated aromatics, and fatty acid derivatives. Polyketide precursors are derived from the conserved polyketide pathway comprising two cytochrome P450 fatty acid hydroxylases, an acyl-CoA synthetase, a type III polyketide synthase (PKS), and a reductase (Grienenberger and Quilichini, 2021). The type III PKS, also known as anther-specific chalcone synthase-like (ASCL), has been shown to be conserved in all embryophytes that produce sporopolleninous exines, including *P. patens* (Suh and Ashton, 2022). ASCL mutants of *P. patens* do not produce the exine and the perine assembles in an irregular fashion (Daku et al., 2016). The hypothesis of conserved biosynthesis of sporopollenin in land plants is generally accepted, although only a few *P. patens* genes (and none from other bryophytes) have been characterized to support the hypothesis.

With few exceptions, the exine is typically deposited on TPL. The exine in liverworts, hornworts, and seed plants is derived from the spore cytoplasm and consequently is constructed from the outside to the inside (centripetally) with the most recently produced wall material on the inside. In many mosses and pteridophytes, the exine is primarily formed by the accumulation of sporopollenin on the outside of existing walls (Lugardon, 1978; Lugardon, 1990). The observation that the exine in *P. patens* forms outside the first spore wall layer, the foundation layer, led Wallace et al. (2011) to speculate that the exine is not deposited by the spore cytoplasm. Our observations that a well-developed exine forms on SMC wall remnants, which connect the adjacent spores and are not in contact with the spore cytoplasm, provide support for the contention that there is an extrasporal contribution to the exine in *P. patens,* but they do not preclude there also being a biochemical contribution from the spore cytoplasm.

Spore wall construction in *P. patens* begins with the deposition of one or two TPL derived from the spore cytoplasm. In bryopsid mosses, this foundation layer typically consists of one TPL impregnated with sporopollenin and forms the region between the exine and the intine (Brown and Lemmon, 1990). The homogeneous exine layer is subsequently deposited on the outside of the foundation layer. Our developmental evidence identifies the exine in *P. patens* as a continuous homogeneous layer of uniform thickness (except over the aperture) that gives a structure to the spore as it expands in the early tetrad and delineates the free spore during the construction of the spore wall. Since the perine is deposited on top of the exine, the moss exine is not the outermost spore wall layer. The exine of mosses may or may not enter into the structure of the ornamented sculptoderm. The exine may serve as a layer upon which the sculptured perine is constructed as seen in *P. patens*. In contrast, in mosses such as *Polytrichum*, *Takakia,* and *Astomum*, the exine forms the base of the sculptural element, while in others, e.g., *Archidium, Ephemerum,* and *Bruchia*, the exine forms the entire element, which is further coated with perine (McClymont and Larson, 1964).

### Perine

Unlike spores of most liverworts and hornworts that develop within the confines of the SMC wall (Renzaglia et al., 2015a; Renzaglia et al., 2015b; Renzaglia et al., 2017), *P. patens* spores expand beyond the sporocyte wall as they mature, leading to the separation of spores from tetrads prior to perine deposition. From this point, the perine accumulates progressively and is responsible for the spore wall ornamentation in *P. patens* as it is in most mosses (Brown and Lemmon, 1990). The deposition of the characteristic spiny sculptoderm occurs in sync with spore expansion as large sporopollenin globules in the capsule locule adhere to specific sites on the spore wall, forming the broad bases of the spiny elements. Further adherence of small sporopollenin granules results in the attenuated tips of the spines. The resistance to acetone treatment, which extracts oil and labile phospholipids (Uddin et al., 2019), and subsequent acetolysis, which removes all non-sporopollenin contents of the sporoderm (Hesse and Waha, 1989), identifies sporopollenin as a constituent of the perine spine. However, this does not preclude the possibility that other biopolymers are complexed with the perine sporopollenin (Neidhart, 1979).

Aya et al. (2011) noted the high expression of PpCYP703B2, which encodes one of the two P450 hydroxylases in the sporopollenin biosynthesis pathway, in “spore sac cells of the sporangium” and suggested the tapetal origin of perine sporopollenin precursors. An ABC transporter protein (ABCG15) is proposed to play a role in the transport of sporopollenin precursors in rice (Qin et al., 2013), and the *P. patens* genome has a single homolog (Pp1s138_50V6) of this protein. According to the *Physcomitrella* eFP browser (http://bar.utoronto.ca/efp_physcomitrella), Pp1s138_50V6 is highly expressed in green sporophytes in agreement with the proposed role. Different subtypes of plant non-specific lipid transfer proteins (nsLPTs) are also involved in the transport of sporopollenin precursors from the tapetum to the microspore exine layer. One particular subtype, type G nsLTPs, is present in mosses and liverworts (Edstam et al., 2011; Edstam and Edqvist, 2014).

### Tapetum

As in anthers and sporangia of other land plants, the tapetum in *P. patens* comprises cells that line the locule in which spores and columella cells develop (Gambardella et al., 1993). Nourishment of spores has been identified as the primary function of the tapetum across land plants (Pacini et al., 1985). The tapetum of angiosperms provides proteins, lipids, polysaccharides, and the sporopollenin precursors required for pollen maturation and construction of their characteristic walls (Zhu et al., 2011). During pollen development, major changes occur in the tapetum as it secretes materials and undergoes programmed cell death (Papini et al., 1999). Similar changes occur in the tapetal inner capsule wall (outer spore sac) and columella cells of *P. patens*, starting with capsule expansion and culminating in internal degradation and death of the cells with the cell walls remaining intact. As the capsule expands prior to meiosis, the inner capsule wall cells, columella, and sporogenous tissue work collectively to produce the locular matrix and interspersed materials, presumably including nutrients, enzymes, and the precursors of sporopollenin synthesis. This metabolic collaboration of sporangial tissue and sporogenous cells leads to the suspension of nascent tetrads in a fibrous network and the simultaneous appearance of sporopollenin globules throughout the locule. Although electron-dense globules partially coat the inner capsule wall cells, there is never a gradation in the abundance of these structures from the tapetum to spore surface, counter-indicating a continuous and steady production and transport of polymeric sporopollenin from the tapetum. We suggest that, in *P. patens,* the biochemical machinery for perine production is present within the matrix of the locule by the time the capsule is fully expanded. We postulate that the majority of cells comprising the capsule, including those of the inner layer of the capsule wall and columella, synthesize and secrete the building blocks for cell wall development in a temporally regulated and collaborative fashion as proposed by Lopez-Obando et al. (2022). Gambardella et al. (1993) similarly concluded that tapetal cells of the bryopsid moss *Timmiella* are involved in spore nourishment and development, but they are not responsible for polymeric sporopollenin secretion.

### Intine

During the development of the *P. patens* spore wall, the intine is initially formed in a patchy uneven fashion, first at the aperture and later around the distal spore beneath the foundation layer. In the mature spore, the intine is a broad and evenly thickened layer that expands in the aperture region. When spores germinate and cells emerge from the spore wall, they rupture the exine but remain surrounded by the intine that is continuous with the newly formed primary walls of the sporeling (Bhandari, 1984; Park and Twell, 2001). It follows that the primary cell wall and intine have the same polysaccharide constituents, namely, cellulose, pectins, and hemicelluloses (Renzaglia et al., 2020).

### Evolutionary considerations

**FIGURE 10:**
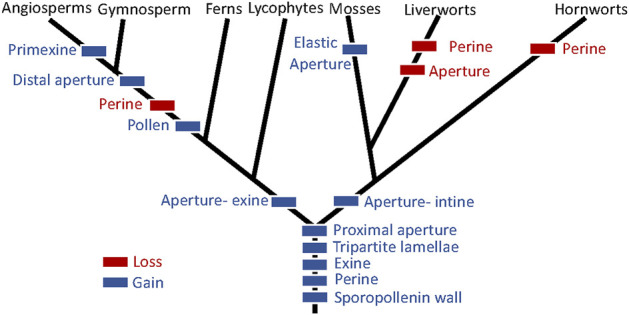
Evolution of spore and pollen wall features based on bryophyte and tracheophyte monophyly and the setaphyte hypothesis (Puttick et al., 2018; Renzaglia et al., 2018).

Comparison of *P. patens* spore structure and development with those of other bryophytes and land plants sheds light on spore modifications that occurred during plant diversification. Since Wallace et al. (2011) assessed the evolution of spore wall development, phylogenetic analyses have converged on a different conclusion, namely, both tracheophytes and bryophytes are monophyletic and mosses are sister to liverworts in a clade referred to as the setaphytes (Renzaglia and Garbary, 2001; Puttick et al., 2018; Renzaglia et al., 2018). This topology is characterized by a primary dichotomy at the onset of land colonization with the divergence of a bryophyte and a tracheophyte clade (Figure 10). Analyses of character evolution based on this topology provide new interpretations of changes in spore wall features over evolutionary time. We will not discuss the intine in this context because it is interpreted, herein, as a modified primary cell wall.

Integrating the observations presented here with data from the literature, we conclude that the earliest land plant spores were surrounded by a sporopollenin-containing wall composed of two layers, an exine containing TPL and a perine that was extrasporal in origin. Early spores possessed a proximal aperture with an expanded exine in tracheophytes and expanded intine in bryophytes. Loss of the aperture and perine occurred in the liverwort progenitor and the loss of perine in hornworts. The elaboration of the elastic aperture as described in *P. patens* is an autapomorphy of mosses. In tracheophytes, the perine is found only in lycophytes and ferns, which also possess proximal apertures. In seed plant pollen, the perine is lost and the exine (derived from both the spore cytoplasm and the tapetum) forms the sculptured surface. Pollen evolution in angiosperms led to great diversity in the pollen structure, variability that reflects the diverse pollination syndromes, and the unique germination and critical function of pollen in the flowering plant life history (Walker, 1974; Blackmore et al., 2007).

Genetic studies on bryophyte sporogenesis have been lagging. With the orchestrated effort to sequence a wide representation of bryophyte genomes through the GoFlag Initiative and 1KP Transcriptomes Initiative, it will be possible to explore the evolution of genes related to the development of the unique structures and processes associated with plant sporogenesis. A particular interest is assessing the occurrence and evolution of sporopollenin biosynthesis genes and tapetal transcription factors. Such bioinformatic analyses will reveal the conservation and/or modification of sporogenesis-related genes along the evolutionary path from spore- to pollen-producing plants, and will provide insight on the genetic control of the development of these structures that are so fundamental to the survival and diversification of plants on land. The morphological information presented in this study provides the developmental details to assess gene evolution and to evaluate phenotypes of sporogenesis-related mutants as they become available.

## Data Availability

The raw data supporting the conclusion of this article will be made available by the authors, without undue reservation.
